# Oxytocin and cholecystokinin secretion in women with colectomy

**DOI:** 10.1186/1471-230X-4-25

**Published:** 2004-10-07

**Authors:** Bodil Ohlsson, Jens F Rehfeld, Mary L Forsling

**Affiliations:** 1 From the Department of Medicine, University Hospital, S-205 02 Malmö, Sweden; 2 Department of Clinical Biochemistry, Rigshospitalet, 2100 Copenhagen, Denmark; 3 Neuroendocrine Labs, Guy's King's and St Thomas Schools of Medicine, SE1 1UL London, United Kingdom

## Abstract

**Background:**

Cholecystokinin (CCK) concentrations in plasma have been shown to be significantly higher in colectomised subjects compared to healthy controls. This has been ascribed to reduced inhibition of CCK release from colon. In an earlier study CCK in all but one woman who was colectomised, induced release of oxytocin, a peptide present throughout the gastrointestinal (GI) tract. The aim of this study was thus to examine if colectomised women had a different oxytocin response to CCK compared to healthy controls.

**Methods:**

Eleven women, mean age 34.4 ± 2.3 years, who had undergone colectomy because of ulcerative colitis or constipation were studied. Eleven age-matched healthy women served as controls. All subjects were fasted overnight and given 0.2 μg/kg body weight of CCK-8 i.v. in the morning. Samples were taken ten minutes and immediately before the injection, and 10, 20, 30, 45, 60, 90 and 120 min afterwards. Plasma was collected for measurement of CCK and oxytocin concentrations.

**Results:**

The basal oxytocin and CCK concentrations in plasma were similar in the two groups. Intravenous injection of CCK increased the release of oxytocin from 1.31 ± 0.12 and 1.64 ± 0.19 pmol/l to 2.82 ± 0.35 and 3.26 ± 0.50 pmol/l in controls and colectomised women, respectively (p < 0.001). Given the short half-life of CCK-8 in plasma, the increased concentration following injection could not be demonstrated in the controls. On the other hand, in colectomised women, an increase of CCK in plasma was observed for up to 20 minutes after the injection, concentrations increasing from 1.00 ± 0.21 to a maximum of 1.81 ± 0.26 pmol/l (p < 0.002).

**Conclusion:**

CCK stimulates the release of oxytocin in women. There is no difference in plasma concentrations between colectomised and controls. However, colectomy seems to reduce the metabolic clearance of CCK. The hyperCCKemia in patients who had undergone colectomy is consequently not only dependent on CCK release, but may also depend on reduced clearance.

## Background

The gut hormone cholecystokinin (CCK) is synthesised in endocrine I cells in the mucosa of the upper small intestine [1] and is released into the blood after ingestion of fatty and protein-rich meals [2]. CCK has various effects on the gastrointestinal (GI) tract and acts on afferent vagal nerves [3], neurons of the myenteric plexus [4], and directly on muscle cells [5]. It is also synthesised in central neurons including hypothalamic, oxytocinergic neurons [6]. Circulating CCK is degraded in several sites, namely the kidney, liver and gut [7,8].

Oxytocin is synthesised in the supraoptic and paraventricular nuclei of the hypothalamus as part of a larger precursor polypeptide [9]. While the main effects are in the myoepithelial cells and uterine smooth muscle in the responses associated with the milk ejection reflex and parturition, the possibility has been raised that oxytocin also contributes to control of the GI motility [10,11]. Both exogenous and food-stimulated endogenous CCK stimulates the pituitary secretion of oxytocin in the rat through CCK-receptors on afferent vagal neurons [12]. In hypothalamus, both parvocellular neurons projecting to the dorsal vagal complex, and magnocellular neurons projecting to the pituitary, secrete oxytocin in response to CCK [13].

We have recently found that CCK also leads to oxytocin release in healthy women [14]. However, one of the women included was colectomised, and she was the only one who had no release of oxytocin in response to CCK [14], although colectomy leads to higher concentrations of CCK in plasma [15-17]. We have found mRNA for oxytocin and its receptor throughout the GI tract [18], as well as the fully expressed proteins (unpublished observation). We do not know if this has an autocrine and/or paracrine role in the gut, or if it also is released into the blood as a hormone. The aim of this study was therefore to examine if women who had performed a colectomy, had a different oxytocin response to CCK than otherwise healthy women with an intact GI tract.

## Methods

### Subjects

Eleven women from the Departments of Medicine and Surgery at Malmö University Hospital (mean age 34.4 ± 2.3 years, range 22–42 years) were studied. They had all a history of colectomy. Two were colectomised because of slow transit constipation (STC) and had undergone a subtotal colectomy with the creation of ileo-rectal anastomosis. Proctocolectomy with ileal pouch-anal anastomosis had been performed in seven of them because of ulcerative colitis, and one because of familial multiple polyposis. The last patient has an ileostomy after subtotal colectomy, saving the rectum, because of ulcerative colitis. Thus, the subjects were cured from their original conditions. The time interval between the proctocolectomy/colectomy and this study was 10–149 months, with a mean of 48.5 ± 12.6 months. Eleven age-matched healthy women with preserved GI tract served as controls. Physical examination and laboratory routine screening were all within normal limits in both groups. The body weight was 68.7 ± 5.8 kg in the patients and 73.8 ± 6.1 kg in the controls. No drugs and no oral contraceptives or other hormonal treatments were allowed in either group. The experiments were performed at no specific stage of the menstrual cycle. None of the included subjects had participated in our former study [14].

### Protocols

The protocols were approved by the local Ethics committee at the University of Lund, and written informed consent was obtained from all subjects before the study was started. The possibility of pregnancy was excluded in all women.

### Experimental procedure

All subjects were fasted overnight. In the morning they were given 0.2 μg/kg body weight of cholecystokinin octapeptide (CCK-8) (Clinalfa, Switzerland) as an intravenous injection. This bolus was chosen as it was the only dose giving raise to a weak, but not significant, oxytocin release in an earlier study [19]. Blood samples were taken through an intravenous catheter 10 min before and immediately before the injection, and 10, 20, 30, 45, 60, 90 and 120 min after the injection.

### Hormone analysis

All blood samples consisted of 8.0 ml whole blood drawn into iced heparinised tubes. The plasma was separated and frozen at -20°C immediately after the experiment. Oxytocin was measured as described by Balment et al [20] using the Fourth International Standard for oxytocin (76/575). The lower limit of detection for this assay was 0.1 pmol/l with intra-assay and interassay variations of 5.4 and 11.8 %, respectively, at 2.5 pmol/l. The hormone was extracted from plasma using C 18 Sep Pak Columns (Waters Associates Ltd., Northwick, Middx., U.K.). The concentrations of CCK in plasma were measured using a highly specific and accurate radioimmunoassay as previously described [21]. The limit of detection for his assay is 0.1 pmol/l with intra-assay and interassay variations of less than 5 % and 15 %, respectively, at both 3.7 and 15 pmol/l concentrations.

### Statistical analysis

The values are expressed as mean ± standard error of the mean (SEM). The basal value is the mean of the two fasting values. The peak value is the mean of the highest concentration in every subject after the injection. The total plasma CCK and oxytocin response was assessed by calculating the area under the plasma concentration time curve (AUC). The Kruskal-Wallis followed by Wilcoxon signed ranks test were used for assessment of the significance of the differences within and between the two groups. The Spearman rank test was used for calculating the correlation between CCK and oxytocin concentrations in plasma. Probabilities of less than 0.05 were considered significant.

## Results

### Plasma oxytocin concentrations

**Figure 1:**
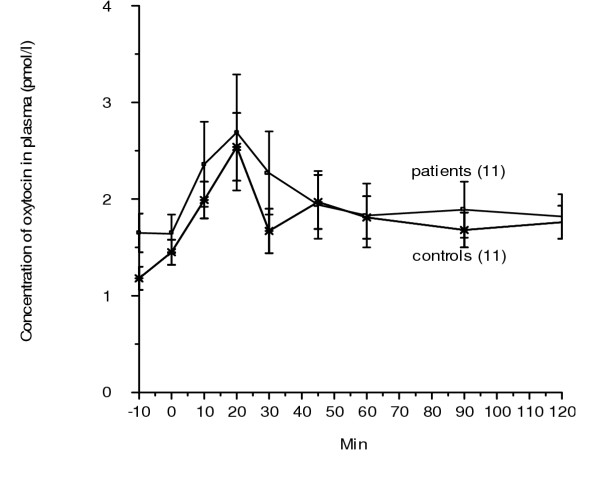
The plasma concentration of oxytocin before and at different time points after an injection of 0.2 μg/kg body weight of cholecystokinin-8 (CCK-8). There were 11 subjects in each group. Values are given as mean and standard error of the mean (SEM). There was no difference between the groups neither when calculating values at different time points studied nor the area under the curve (AUC). Wilcoxon signed rank test.  = control, = patient.

The basal oxytocin concentration in plasma was similar in the two groups. The concentration was stable before the start of the experiments. Injection of CCK-8 led to an increase of the oxytocin secretion compared to basal values in both groups (p < 0.001) (Table 1). The increase in plasma concentration of oxytocin was observed after 10 min and persisted throughout the study. The highest concentration was found after 20 min (Fig 1). There was no difference of the AUC between the two groups, neither there was any difference between each time point studied (Fig 1).

**Table 1:** Basal and peak plasma values of cholecystokinin (CCK) and oxytocin

	CCK (pmol/l) N = 11	Oxytocin (pmol/l) N = 11
Controls		
Basal	0.7 ± 0.1	1.3 ± 0.1
Peak	0.9 ± 0.1	2.8 ± 0.4***
Patients		
Basal	1.0 ± 0.2	1.6 ± 0.2
Peak	1.8 ± 0.3**+	3.3 ± 0.5***

Values are expressed as mean value ± standard error of the mean (SEM). Comparisons are made within groups; ** = p < 0.01, *** = p < 0.001, and between groups; + = p < 0.05. Wilcoxon signed rank test. The basal value is the mean of the two fasting values. The peak value is the mean of the highest concentration in every subject after the injection.

### Plasma cholecystokinin concentrations

**Figure 2:**
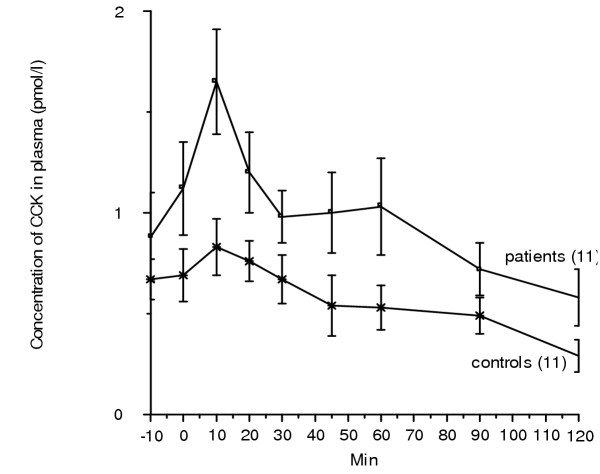
The plasma concentration of cholecystokinin (CCK) before and at different time points after an injection of 0.2 μg/kg body weight of CCK-8. There were 11 subjects in each group. Values are given as mean and standard error of the mean (SEM). When calculating the area under the curve (AUC), there was a significantly increased AUC in the colectomised subjects compared to controls (p < 0.04). No difference was seen between the groups when comparing values at each time point. Wilcoxon signed rank test.  = control, = patient.

There was a tendency towards higher basal CCK concentration in patients, although not significant (Table 1 and Fig 2). CCK-8 has a half-life in plasma of about < 1 min (8). Therefore, no increase in plasma CCK could be detected in the control group after the intravenous injection of CCK-8 (Table 1). However, in colectomised women, an increase in plasma CCK concentrations was found after the injection compared to basal values (p < 0.002). The difference of peak value between the groups was significant (p < 0.04) (Table 1). The AUC differed significantly between the two groups (p < 0.04), but no difference was observed between values at each time point studied (Fig 2).

There was no correlation between CCK and oxytocin concentrations (data not shown). Neither was there any difference in CCK and oxytocin concentrations between patients with different diagnosis and those who had rectum saved or resected (data not shown).

## Discussion

This study shows for the first time that CCK-8 increases the secretion of oxytocin in women. We have previously shown that exogenous CCK-33 and -39, and a fatty meal with endogenous CCK release, led to enhanced concentrations of oxytocin in plasma [14]. One patient in that study was colectomised, and she was the only one in whom no increase in oxytocin release was seen after CCK stimulation. This observation prompted the present study. In this study, there was no difference in plasma concentrations of oxytocin between colectomised and healthy controls. Thus, the oxytocin secreted into the blood after CCK stimulation seems not to origin from the colon. The oxytocin recently found in the colon may participate in autocrine and/or paracrine regulation of the gut while having no endocrine effects [18]. The patient group examined in this study was not homogenous, but it was not possible to include enough young women with colectomy after ulcerative colitis. We have earlier described the presence of oxytocin and its receptor throughout the gut, without any efforts to quantify the expression [18]. Only the effect of colon on plasma concentrations of oxytocin was measured in the present study. We do not know from this study if oxytocin from some other part of the gut is released into the plasma. It is difficult to conduct an experiment to examine the origin from the oxytocin release. CCK acts on receptors on afferent vagal nerves to stimulate the oxytocin release from the pituarity [12], and these receptors are present throughout the GI tract [3-5]. Therefore it is not possible to use CCK-receptor antagonists to distinguish between central or local CCK effects.

CCK has been shown to stimulate oxytocin secretion in mammals in many studies [12-14]. However, in a previous study, intravenous injection of CCK-8, in the same dose as in our study, did not increase the concentration of oxytocin in plasma [19]. This may depend on methodological differences. Another possible explanation to the difference is the effect of gonadal hormones on the regulation of the oxytocin release from the posterior pituitary gland. In our study, only women were included, whereas Miaskiewicz et al [19] examined 13 men and one woman. Orally administered estrogen stimulates oxytocin secretion, and progesterone also affects release [22]. Lower plasma levels of these hormones in men may explain the absence of increased oxytocin secretion in men. In addition, one study has shown that testosterone inhibits the secretion of oxytocin from the pituitary gland [23].

Oxytocin is present in plasma in men, although at lower concentrations [24,25], and shows a circadian rythm [26]. Oxytocin may have similar effects on the GI tract in men and women, although the plasma concentrations differ. The effects of oxytocin have been only rudimentary examined. However, oxytocin has in one study been shown to enhance gastric emptying [10], and in a yet unpublished study, we have found that an oxytocin-receptor antagonist delayed the gastric emptying rate (unpublished observation). Further, we have demonstrated increased colonic peristaltis after oxytocin stimulation in healthy women [11]. Our finding of oxytocin release in response to a meal [14], and the presence of oxytocin receptors on the cells that regulate the gut motility (unpublished observation), suggest oxytocin to play a physiological role in the GI function.

Several studies have reported that after colectomy in different species there are higher concentrations of CCK in plasma, both basal and postprandial, compared to healthy controls [15-17]. It has been suggested that this is due to depletion of an inhibitory factor of CCK secretion which is released from the colon. Peptide YY (PYY) is secreted from distal ileum and colon, and CCK is known to stimulate PYY secretion from the hindgut [27-29]. PYY then inhibits further CCK secretion [30,31]. As PYY is secreted from the hindgut, this peptide is substantially reduced after colectomy [26]. Thus, the reduced PYY concentration may explain the hyperCCKemia. In this study, the elevated CCK concentrations in the group of colectomised women were not due to increased secretion of CCK, as CCK was injected exogenously. Instead, the hyperCCKemia in the group of colectomised patients seems to be due to reduced degradation of the peptide injected. CCK is degraded in the kidney, liver and gut [7,8]. Our hypothesis is that PYY could contribute to the degradation as well as the secretion of CCK. Receptors for PYY have been found in the kidney and on hepatocytes, and PYY influences the renal and hepatocyte metabolism [32-34]. Alternatively, the different CCK concentrations could be due to reduced degradation in the colon in addition to the kidney and liver, as CCK-8 has been shown to be degraded in the gut in pigs [8]. It remains to be determined which mechanism contributes most to the hyperCCKemia observed after colectomy; increased CCK secretion, or decreased clearance. In the present study, the basal levels of CCK did not differ significantly in colectomised, as observed in earlier studies [15-17].

CCK has a wide range of effects on the GI tract. Three physiological effects on gut motility have been identified; contraction of the gallbladder [2], relaxation of the sphincter Oddi [35] and inhibition of gastric emptying [2]. Its role on colonic motility is controversial. While Barone et al [36] were able to demonstrate contractions, Niederau et al could find no effect of CCK [37]. Further, CCK increases pancreatic enzyme secretion [2]. It is not known if the hyperCCKemia observed in colectomised patients [15-17] has any impact on GI motility or health.

## Conclusions

CCK stimulates the release of oxytocin in women, probably via an effect on the neurohypophysial system. There is no difference in plasma concentrations between colectomised women and women with intact GI tract. The hyperCCKemia observed in patients who have undergone colectomy is dependent not only on an increase in CCK release, but may also depend on a reduced degradation. It was beyond the aim of our study to determine the clearance of CCK. However, this should be evaluated further.

## Abbreviations

AUC = area under the curve

CCK = cholecystokinin

GI = gastrointestinal

PYY = peptide YY

SEM = standard error of the mean

## Authors' contributions

BO designed the study, included patients, paid for the most, performed the statistical analysis and drafted the manuscript

JR carried out the radioimmunoassay for CCK and participated in the writing process

MF carried out the radioimmunoassay for oxytocin and participated in the writing process

All authors read and approved the final manuscript.

## Competing interests

The authors declare that they have no competing interests.

## Pre-publication history

The pre-publication history for this paper can be accessed here:


